# DEPRESSIVE SYMPTOMS AND HANDGRIP STRENGTH IN MEXICAN AMERICAN OLDER ADULTS: SEX DIFFERENCES

**DOI:** 10.1093/geroni/igae098.3946

**Published:** 2024-12-31

**Authors:** Jamil Okada, Joshua Patino, Ariza Martinez, Soham Al Snih

**Affiliations:** UTMB, Bedford, Texas, United States; The University of Arizona, Tucson, Arizona, United States; The University of Arizona, Tucson, Arizona, United States; University of Texas Medical Branch in Galveston, Galveston, Texas, United States

## Abstract

The aim of this study was to examine the role of sex in the relationship between depressive symptoms and handgrip strength decline among Mexican American older adults aged ≥65 who scored normal or high in handgrip strength (≥17 Kg in females and ≥27 Kg in males) at baseline over 23 years of follow up. Participants (N=1226) were from the Hispanic Established Population for the Epidemiologic Study of the Elderly (1993/1994-2016). Measures included high depressive symptoms as the independent variable assessed with the Center for Epidemiological Study Depression Scale (CES-D ≥16), handgrip strength in kilograms as outcome variable, and socio-demographics, comorbidities, body mass index, cognitive function, physical function, and disability as covariates. General linear mixed models were used to estimate the population average change in handgrip strength as a function of high depressive symptoms over time. Female participants with high depressive symptoms experienced a greater [β= -1.34, Standard Error (SE)= 0.26, p-value= < 0.0001] decline in handgrip strength over time than females without high depressive symptoms after controlling for all covariates. No significant decline (β= - 0.52, SE= 0.39, p-value= 0.1812) in handgrip strength as a function of high depressive symptoms was found among male participants over time after controlling for all covariates. In conclusion, we found that the effect of high depressive symptoms was greater among female than male participants. Screening and treatment of depressive symptoms may delay decline in muscle strength to further delay disability, maintain independence, prevent institutionalization, and reduce the risk of mortality in this underserved population.

